# "The Hospital" Nursing Mirror

**Published:** 1901-09-21

**Authors:** 


### "The Hospital" Nursing Mirror.

Being the Nursing Section of "The Hospital."

[Contributions for this Section of "The Hospital" should be addressed to the Editor, "The Hospital" Nursing Mirror, 28 & 29 Southampton Street, Strand, London, W.C.]

### Notes on News from the Nursing World.

### "I WANT TO SEE THE TREES."

It appears that no less than six nurses were in attendance upon Mr. McKinley at the time of his death. This shows that great care was taken not to overtax them, although Mrs. McKinley, in her delicate state of health, frequently required assistance. Only two of them seem to have been actually in the patient's room at the same time, except about ten minutes before the end, when all assembled in the circle round the unconscious sufferer. The sole request which we are told the President preferred to them is likely to be long remembered by all who have read the record of his closing hours. On the last day he was conscious and two of the nurses sought to adjust his pillows, so as to shut out the light from the window, he protested, and murmured, "No, I want to see the trees, they are so beautiful."

### OUR CHRISTMAS DISTRIBUTION.

No apology, we are sure, is needed from us for again drawing the attention of our readers to our Christmas distribution. Each year the number and variety of useful articles of clothing made with kind hands by the possessors of kind hearts for the use of the patients in hospitals and infirmaries increase, but so also do the demands. Last year, thanks to the very liberal response to our appeal, we were able to send parcels to institutions which had not previously received them, and we hope, during the coming Christmas season, to make yet further additions to our list. If every nurse who can help in any way will forward us something, the refusals which we are regretfully obliged to return, even to pressing applications, will be comparatively few. The name and address of the sender of each parcel should be enclosed, in order that the gift may be acknowledged in our columns; and all parcels should be addressed, The Editor, *The Hospital*, 28 & 29 Southampton Street, Strand, London, W.C. The sooner they reach us in December the better; but the latest date is Monday, December 16th.

### RETURN OF NURSES FROM SOUTH AFRICA.

The *Nubia*, which left Natal for England on September 12th, includes among her passengers the following nurses who are invalided home:—Sisters Palmer, Gordon, Nutter, Phillips, Beetham, Douglas, Rider, Wells, and Hunsellin. The *Dilwara*, which arrived at Southampton on Monday, had on board Sisters A. M. Glennie, A. Hill, and M. L. Harris, who all return to South Africa on expiration of leave.

### AN ENGLISH SISTER IN THE REFUGEE CAMPS.

A nursing sister who has been for nearly six months engaged at the hospital of the refugee camp at Aliwal North emphatically dissents from the reports circulated by Miss Hobhouse regarding the condition of the refugee camps. Miss Headland, whose communication was passed by the press censor, justifies her own view that the refugees are treated with great consideration by the simple recitation of convincing facts. "Everything possible," she says, "is done to make them happy. They have tennis courts, croquet, football, and golf to amuse them; school for the children; a resident clergyman who holds Divine service both in Dutch and English. The food is good but plain. Every adult is allowed 1 lb. of meat per day. Tea, coffee, sugar, milk, and salt are issued out weekly, quite sufficient. At one time bread was issued, but they constantly complained about it and now meal is given instead. The bread was very good, not coarse. We are receiving the same in hospital now, and could not wish for better. Thousands in England would be only too pleased to get half of what these people get." Miss Headland does not conceal her opinion that some of the refugees by no means merit the excellent food and attention they receive at the hands of our officials.

### NURSING TYPHOID AT COVENTRY.

The nurses attached to the Coventry Nursing Institution, of which Miss L. A. Wing is lady superintendent, are winning golden opinions in the town by their devotion to duty under very difficult conditions. The bedrooms available in the homes where typhoid has attacked the inmates are only two, or three at the outside, and in several instances where most of the family are suffering from the disease the patients have been compelled to share the beds. In one case the whole of a family of eight were down with it, three children occupying one bed. The character of the epidemic may be judged from the fact that five nurses have been in constant attendance upon the patients in a single street. One of these has, unfortunately, broken down, and, though not a victim to typhoid, will not be able to resume her duties for some time. It is gratifying to learn that the townspeople are responding liberally to Miss Wing's appeal for assistance, and that the isolation accommodation, whose urgent need the outbreak has demonstrated, is in course of preparation.

### THE IRISH PROVINCIAL TRAINING SCHOOLS.

The question whether the provincial hospitals in Ireland are in a position to carry out an efficient training is raised by the medical officer to the Fermanagh Infirmary. He affirms that, except in the cases of a few which are specially favoured by intelligent management to bring and keep them up to a modern standard, it must be admitted by all hospital experts that the union hospitals and many of the county infirmaries are not adapted to treat the patients according to modern standards, to say nothing of training nurses in modern methods. He confesses that before the extensive alterations which have taken place during the last two years at Fermanagh Infirmary itself, he should have had no hesitation in condemning it as a school for training nurses; and he states that in several county infirmaries with a less enterprising committee of management, the reply made to the surgeons' representation as to the unfitness of things is, "Oh, it has done well enough for the last sixty or seventy years; it is good enough." Against this view, which in effect prevailed at the recent conference, he protests, and urges that the Irish Local Government Board should be asked by the Poor Law Guardians to have both the county infirmaries and the union hospitals inspected and reports issued by the inspectors on their fitness for training in modern methods, their equipment, the facilities offered for teaching probationers, and the examinations. He further suggests that these inspections having taken place, the Local Government Board should accept only certificates of training as proof of efficiency from those institutions where all the conditions are satisfactory. This is a practical way of looking at the matter, and we rejoice to see that Dr. Kidd expresses the hope that the Local Government Board will not yield "to any crude scheme of accepting the training of all provincial hospitals as satisfactory." If resident medical officers generally in the sister island will adopt this attitude, they will do their part towards elevating the standard of nursing in the workhouse infirmaries.

### LEAVE OF ABSENCE AT ARMAGH.

The Armagh Board of Guardians and the medical officer are indignant because the Irish Local Government Board have found fault with the medical officer for allowing the infirmary and the fever hospital to be without a qualified nurse in charge. The defence of the latter was that on each occasion when he granted leave of absence there were no cases either in the infirmary or the fever hospital which required constant nursing supervision; and, as might have been expected, the Armagh Guardians, who cannot forgive the Local Government Board for resolving to improve the nursing in workhouse infirmaries, found it satisfactory. But the inspector whose report prompted the Local Government Board to a remonstrance, did his duty in calling attention to the matter. Under no circumstances should a number of patients, any of whom may at any moment develop symptoms needing immediate attention, be left unattended by a trained nurse. The demand of the guardians that the Local Government Board should withdraw their animadversions on the medical officer is not, we imagine, at all likely to be conceded.

### ENGAGEMENT UPON TESTIMONIALS.

The Gainsborough Board of Guardians are experiencing a difficulty in obtaining nurses for the workhouse infirmary. The clerk, who is of opinion that good nurses have been lost because of the practice of requiring applicants to appear before the Board and the consequent delay, has therefore advised the Guardians to engage entirely upon the testimonials received, and this suggestion has just been adopted. The result of the change in policy will be watched with much interest. While it is true that time may be saved by it, there are advantages even from the nurse's point of view, in the arrangement which has been discarded. If she has to undergo the ordeal of being catechised by guardians who often interlard irrelevant questions with stupid jokes, she is at any rate thus afforded the opportunity of learning something about the conditions under which she will have to work. The proper plan would, of course, be to invest the superintendent nurse with authority to appoint or discharge her own staff; but under the present anomalous system, engagement upon testimonials may possibly answer better than engagement after a personal interview with a number of men who have not the special knowledge necessary.

### THE RESIGNATIONS AT CROYDON INFIRMARY.

In another column will be found the official announcement of the resignation of half a score of the nurses of Croydon Infirmary. We observe that one of the guardians calls attention to a statement which, he says, appeared in "The Hospital" Nursing Mirror to the effect that "13 or 14 of the nurses had resigned lately." No such statement was ever made in this paper. On August 31st we intimated the resignation of six nurses, and a correspondent in our issue of last week assured us that five more resignations were impending. As 10 have actually taken place, our information was not far wide of the mark. The Chairman of the Infirmary Committee practically stopped discussion on the subject at the meeting of the guardians on Tuesday, and we do not, therefore, know their view of the present position. But we fancy that the medical superintendent will have to avail himself pretty largely of the permission vouchsafed to him, "to engage temporary nurses to fill the vacancies."

### THE HOURS OF NURSES.

While it is true that the hours of nurses in some hospitals may be too long, we are glad that "A Nurse's Mother," who sends us an interesting letter on the subject, does not suggest legislation as a panacea for the cure of the alleged grievances of hospital and private nurses. She does, however, commit herself to a statement which would be very serious if its accuracy could be proved. Our correspondent avers that "many probationers do not get beyond their first month's trial," and "that many cannot bear the strain more than six." As a month's trial is purposely given to test the fitness of a candidate for the post of probationer, physical as well as otherwise, the fact that a considerable number do not get beyond it is not surprising, though at least a certain proportion of these are found unsuitable for other reasons than lack of physical strength and stamina. But the assertion that many who are accepted as probationers cannot bear the strain more than six months, is at variance with the experience of the matrons of the leading hospitals, and is, we are persuaded, founded on the most slender material. Our correspondent observes that the ladies who wish to enter the profession are "not young girls," and though 24 is not a patriarchal age, it is quite true that candidates for probationerships are supposed to have attained years of discretion, and to be qualified to judge whether the conditions of work are such as they are able to undertake. They do not start in ignorance of the hours of duty, which are always clearly stated; and if they think that they are excessive, they need not victimise themselves. As to private nurses, who can less appropriately be described as "young girls," our correspondent thinks that the remedy even for hours of duty extending to 140 hours a week, can only arise from a fuller recognition of what is due to a nurse from her employers. But the services of capable private nurses are far too much in request to render it necessary for them to submit to slavery.

### A HOME FOR THE NURSES OF ASTON WORKHOUSE INFIRMARY.

The statement in a Birmingham daily paper in reference to Aston Workhouse Infirmary, cited in our last issue, which we assume was not printed without inquiry, is described by a well-informed correspondent, as opposed to facts, particularly the allegation that "there is no paucity of nurses." The truth, we are assured, is that there are as many nurses as the existing accommodation will provide for, but that several more are needed. The difficulty as to accommodation will be met when the new Nurses' Home, which is approaching completion, is finished. We are glad to learn that whatever may be the lack of nurses now, it is likely to be speedily remedied.

### LIBEL BY A NURSE ON A DAWLISH DOCTOR.

At Dawlish Police Court last week, Clara Cooper, nurse, of 5 Lansdowne Place, was charged with having written and published certain false, scandalous, malicious, and defamatory libels respecting Dr. Charles Newton Lovely, a surgeon in practice in the same town. Mr. Hutchings, who defended the accused, said he did not resist a committal, but he suggested that she was temporarily not responsible for her actions. As he freely admitted that "there was not a word of truth in what she had said," Dr. Lovely has not to wait for the vindication of his character until the trial, which the Bench however considered necessary, and the case will therefore be heard at the county assizes.

### NO MORE SYMPATHY.

In an accident ward in a large Midland hospital a patient suffering from a compound fracture of the tibia frequently complained that he could get no sleep at nights from the pain in his leg. This had no doubt been the case at first, but as the inflammation subsided so did the sleeping capacity of the patient improve. One night the nurse went as usual to change his fomentation, and found him gently snoring. To the great amusement of the patient in the next bed, she carefully turned back the bed-clothes, took off the side splints, and changed the fomentation without waking the patient. Next morning the man began, as usual, to describe what a sleepless night he had passed, but was stopped, and the rest of the ward greatly entertained by an account of how he had been caught and dressed "napping." He did not again enlarge upon his sleepless nights.

### A PENNY PER WEEK.

Although the Barry District Nursing Institute is well supported by the working classes, who last year contributed in subscriptions of a penny per week no less than £400, it is pointed out in the report that this amount might be greatly swollen if all the men employed at the docks would follow the example of the minority. In that case an income of about £1,200 a year would be assured, and it would be easy both to increase the efficiency of the institution and to pay the debt on the new premises of £1,100. As it is, the receipts for the twelve months were £144 in excess of the expenditure; but the executive are naturally anxious to augment the nursing staff, and to feel that the handsome Victoria Jubilee Nursing Home is paid for. It is suggested that the churches and chapels might afford more substantial support than they have hitherto done; but we advise the committee to aim above all things at securing the penny a week from the 6,000 workmen, who will then be in the proud position of saying that they run the association with their own money.

### A QUESTION OF SALARY.

At the meeting of the Chorlton Union Board of Guardians on Thursday last, a recommendation to appoint a matron for the Cottage Homes at Styal at a salary of £50 a year was objected to on the ground of extravagance. But if there is to be a matron at all—and it stands to reason that the homes, which are on a large scale, must be under the charge of some efficient person—the salary offered could hardly be less than £50 a year. We are therefore glad to observe that, in spite of the grumblers, the recommendation was carried. The advantages of the matron in such a position being a trained nurse are obvious.

### THE NURSES' HOME AT HASTINGS.

The Hastings people are very much interested in the new District Nurses' Home, which is now practically completed. It will be under the management of a lady of experience, and the staff will consist of two fully-trained nurses who are also midwives. Their services will be available exclusively for the poor. It is hoped that in time enough money will be raised to extend the work, which is at first to be confined to the Old Town—the district most in need—to the whole of Hastings and St. Leonard's.

### SPORT AND NURSING ASSOCIATIONS.

It is pleasing to hear of cricketers and football players contributing, by means of the proceeds of gate money, to the funds of nursing organisations. At Huddersfield the other day a cheque for £21 16s. 3d. was sent to the secretary of the Victoria Sick Poor Nurses' Association, representing the amount received at a cricket match between the Golf Club and the Professions. The "very valuable" help of the Raunds football club was warmly acknowledged at the annual meeting of the Raunds Nursing Association. As we always insist, the financial mainstay of a nursing organisation ought, of course, to be the annual subscriptions which all classes should combine to make as numerous as possible. But sportsmen, who frequently suffer from accidents on the field, know the importance of skilful nursing; and they could not show their special appreciation of it in a more practical and welcome manner than that happily adopted by the cricketers at Huddersfield, and the football players at Raunds.

### THE "LEICESTER" NURSES' HOME.

Rapid progress is being made with the "Leicester" Nurses' Home at Norwich. The building, which is designed in a style calculated to harmonise with the Norfolk and Norwich Hospital, will contain about seventy rooms, of which sixty will be bed and private rooms assigned to individual nurses. There will also be two general sitting-rooms, a sick dormitory, and apartments for the superintendent and the night superintendent. The home will be lighted throughout by the electric light and provided with every modern convenience, including a commodious bicycle shed. The cost will not be far short of £15,000.

### SAILOR AND SISTER.

An old sailor who entered one of our London hospitals to undergo a slight operation seemed to delight in observing all the rules to the letter, and to any order given he sang out in his cheerful fashion, "Right you are, Nuss!" "No. 5," said sister one day, "Don't say 'right you are;' every time you are told to do anything say 'yes nurse' or 'very well, nurse.'" "Right you are, nuss!" was the ready rejoinder. Then he gasped apologies, but sister only smiled.

### SHORT ITEMS.

The War Office has been notified from South Africa that Nursing Sisters Margaret Evelyn Howell and Katherine J. Cuming Smith were discharged from hospital to duty the week ending September 3rd.—A demonstration on Saturday, September 8th, at Gainsborough, which took the form of a street carnival, realised upwards of £70 for the benefit of the Gainsborough Nursing Institution.—Under the auspices of Mr. George Adney Payne, contributions are being received from music hall artistes to the Women's Memorial to Queen Victoria. A substantial sum has already been subscribed.

### The Preparation of Patients for Anæsthetics and the After Treatment.

By W. A. MILLIGAN, M.A., C.M., F.R.C.S.(Edin.), Anæsthetist, Great Northern Central Hospital.

### I.—PREPARATION.

IN considering the question as to the best mode of preparing a patient for being anæsthetised, a great many factors, peculiar to each individual case, come into play, and so no hard and fast rules can be laid down. At the same time the general principles of preparation always remain the same, and by applying these in an intelligent way, a nurse can do a great deal towards the comfort of her patient, towards lightening the task of the anæsthetist, and thus through him towards the success of the operation. Let us then for convenience and simplicity discuss the mode of preparation under the following heads:—

1. Diet.
2. Regulation of bowels.
3. General preparation.

1. *Diet.*—As to this, there is no question that the proper regulation of the diet is a most important, if not the most important, factor in the preparation of a patient for an anæsthetic. The great point to be remembered is that the stomach should be empty, although at the same time the patient must not be faint from hunger. To ensure success in this direction one must take each case on its own merits, and prepare accordingly. In the case of a healthy adult, whom perhaps the nurse has had the advantage of having under her care for a few days prior to the operation, the preparation should be carried out on these lines:—For the day previous an easily digestible diet should be given, *e.g.*, a diet consisting of soups, fish, fowl, tripe, and the meals given at as nearly as possible the time when the patient is accustomed to have them. The stomach digests better at these times than if food be given at unusual hours.

Now, supposing the operation is to take place at 9 A.M., the patient should be allowed the evening meal as usual, but after that nothing. If, however, the operation is not to be performed until later in the morning, say, 10 to 10.30 A.M., then a small cup of tea with half a slice of toast at 6 A.M. or a cup of clear soup is permissible. In the case of a patient enfeebled by age or disease, too long a fast must not be allowed, and a cup of beef extract or non-greasy beef tea four hours before operation should be given. In addition to this, and especially if the case be in any way an extreme one, a nutrient enema consisting of peptonised milk, beef tea, and brandy (℥i of each) should be given half an hour previous to operation, the rectum having been previously washed out with warm water.

In the case of children the preparation must necessarily vary somewhat. Very young infants, say to six months, should be allowed their usual nourishment two to two-and-a-half hours previous to time of operation. Children, six to 12 months, should be fed on what they are accustomed to have, three hours previous to operation. In those cases of a year and upwards, and this applies also to adults, it is well to remember that milk is not a suitable food to give prior to anæsthetisation. Milk is slow of digestion, and is very apt to come up in curdled masses either during anæsthetisation or after the operation. It is better then that the child should have beef tea, ℥ii–℥iv, according to age, four hours before the operation.

In speaking of diet, a few words might be said with regard to alcoholic stimulation. In the first place then let me say, in preparing a patient for anæsthetisation, alcoholic stimulation should be avoided as much as possible. There are no doubt some cases when it is necessary that some such stimulation should be given, but as a general rule it should be avoided whenever possible, and under no circumstances should whisky or brandy be given by mouth half an hour or an hour before the operation, for the stimulant tends to produce retching and vomiting. If necessary, then let it be given along with a little beef tea four hours before, and should anything more by way of stimulation appear advisable, it should be given in the form of an enema, ℥i brandy in ℥ii beef tea half an hour before operation, the rectum being washed out before it is administered.

2. *Regulation of Bowels.*—This is another very important point in the preparation of a patient before being anæsthetised. The nurse must here again be largely guided by the circumstances of the case. If she has her patient under her charge for a few days prior to the operation, she must regulate the bowels by means of laxative medicines as cascara, senna, etc., following these, if need be, by salines. On the night previous to operation a purgative should, under ordinary circumstances, be given, *e.g.*, castor oil—far the best for children—rhubarb, liquorice. Some advise the purgative being given not one but two nights before, as it is said that this greatly allays tendency to vomiting. I have said that under ordinary circumstances a purgative should be given, but there are cases in which it is not desirable, *e.g.* in debilitated patients, where a purgative may tend to increase any tendency to collapse; or, again, as has been pointed out by Snow, if the operation is to be on the rectal region, a purgative tends to induce syncope during anæsthetisation. As to this, however, the nurse will receive orders from the medical attendant. I merely want to point out that to give a purgative is not always advisable in the preparation of a patient. Whether the treatment adopted be purgative or laxative it should always be followed on the morning of operation by an ordinary soap and water enema, the rectum being subsequently washed out with a little warm water.

So far, what has been said in the matter of preparation of patients applies to those who are to be put under the influence of ether, or chloroform, or some combination of these. Should the case be one for gas, such elaborate preparation is unnecessary, although it is very important that even then a patient should not have anything by way of nourishment for at least four hours previous to operation.

3. *General Preparation.*—On the day of operation the patient must be kept quiet in bed and everything in the way of preparation should be done as quietly and as quickly as possible. If the patient be at all of a nervous temperament the nurse should do all she can to encourage him or her. The clothing should be warm and of such a kind that the whole body is well covered, a pair of long stockings being put ready to slip on before the patient is removed to the anæsthetic room. The clothing must not be tight, especially round neck or chest. If bandages be used to keep any antiseptic compresses in position anywhere in these parts, they should be loosely applied. Absolute freedom of movement for the chest is a very important matter.

Any false teeth must be removed from the mouth, and a careful watch kept that nothing be smuggled into the mouth by the patient. The patient should always pass water before going in to the operation room, and for some operations it is so essential that the bladder be empty that the catheter is ordered to be passed. Children are very apt to void urine unconsciously under the influence of gas, therefore particular attention should be paid to this particular before the administration of gas is begun.

The removal of the patient from bed to the anæsthetic room should always, if possible, be done on a stretcher, and this is essential if the operation is of a serious nature, more especially an abdominal one. In any case the patient should never be allowed to walk upstairs from bed to operating room.

Such, then, are the lines on which a patient should be prepared for anæsthetisation where there is sufficient time to carry them out. It must, however, and often does happen, that a patient has to be got ready hurriedly, and there is not time for much in the way of preparation; but it should be carried out so far as circumstances will permit on the above lines.

In an emergency case, *e.g.*, strangulated hernia, it is well, especially if food has been recently taken or if there is much vomiting, that the stomach should be washed out; therefore all should be got ready for such performance in case it is deemed wise. In any case a good enema should be administered, and after washing out the rectum a nutrient enema should be injected, more especially if the patient has collapsed.

II.—THE AFTER TREATMENT.

In the first place the nurse must see that the bed into which the patient is to be put has been well warmed, taking care, however, that any hot-bottles left in after the patient has been placed in bed are well covered with flannel, as neglect of this may lead to the patient being burned. The temperature of the room should be from 65° to 70° Fahr., and every care taken to guard against the patient getting chilled. The patient must be very carefully watched till the effects of the anæsthetic have completely passed off; the time required for such recovery varying much. The patient must on no account be allowed to sit up. If there is retching and vomiting, a very common occurrence after ether or chloroform administration, and one of the most troublesome things the nurse has to combat, the patient should, if the surgeon permit it, be turned on his right side so that the vomit may escape from the cheek, and not get down the throat. If, however, it is essential that the patient be kept on his back, then the head should be turned to the side and the vomit thus allowed to flow from side of mouth. If there is difficulty in getting the vomited matter away, the chin should be drawn well forwards, and thus the escape facilitated. Should the vomiting be very persistent small sips of very hot water, small pieces of ice to suck, or black coffee, or sips of champagne may be tried. A poultice, mustard and linseed (1–6), placed over the stomach, a mustard leaf at pit of stomach, or a turpentine stupe sometimes give relief. Hypodermic medication has sometimes to be resorted to, but this the nurse would only give by the doctor's orders.

Then again the thirst which patients suffer from, especially noticeable after an abdominal operation, is sometimes very severe and trying. To help to alleviate this, the nurse should get her patient, as soon as he is sufficiently conscious, to wash the mouth well out with warm water. Swabbing out the inside of mouth with glycerine and borax sometimes relieves considerably; sips of soda-water into which a few drops of fresh lemon have been squeezed, small pieces of ice to suck, moistening the lips with brandy and water—any of these may help, but the only real cure for both sickness and thirst is, time.

No nourishment at all should be given by mouth for at least four hours after operation, and even then must be withheld if sickness continue. As in the preparation for operation, so in the after treatment, milk is not very suitable. To begin with, then, a little Brand's meat jelly should be tried and repeated if no sickness is produced, and the patient be thus gradually worked on to light articles of diet. If after the operation there be much collapse, or the vomiting prove intractable, a nutrient enema should be at once administered, and repeated if necessary, and so soon as the stomach will retain, small sips of brandy and water, iced if necessary, should be given at frequent intervals.

Very careful watch must be kept over the condition of the pulse, and if this should get weak, the foot end of the bed should be raised so that the patient's head is low, and thus the tendency to syncope be averted.

Such then are the main points to be attended to in the preparation of a patient for an anæsthetic, and in his or her after treatment many other points, which space forbids us entering upon, will crop up in individual cases, and which the nurse will have to treat, guided by her own common sense and experience.

### The Royal Visit to No. 5 General Hospital, Wynburg.

By A Member of the Army Nursing Service Reserve.

When we returned from Capetown on the day of the arrival of the Duke and Duchess of Cornwall and York, Wynburg seemed a perfect little paradise of rest and quietness after all the noise and glare of the town, but it was only waiting for the following day to play its part in the general rejoicing. For two or three days "Tommy" had been busy making the necessary improvements in his personal appearance. Groups of orderlies might be seen polishing buttons and badges, and any patient who understood the art of haircutting found himself in great demand.

Wasted Decorations.

On Tuesday morning came the news that, contrary to expectation, the Duke and Duchess would go through the wards of the hospital. As soon as this was made known the convalescent patients hurried to the nearest hedges and trees to rob them of their flowers to decorate their wards, and there were many fears lest No. 28 should be smarter than 29 or lest ward 31 should copy 32. Their decorations, however, were doomed not to be admired by the Royal visitors, for at mid-day a fresh order was given out that all patients who were well enough were to parade by the side of the main road, as the Duke and Duchess would only have time to drive through the camp. Fortunately, they were all well enough to walk, or be carried to the spot, with the exception of one enteric who had not yet arrived at the stage of being marked "up."

Arrival of the Visitors.

By 2.30 p.m. medical officers, sisters, and patients were all in their places—the latter resting on the bank—and then commenced a long and weary wait. Though we were thankful for the fine weather, we sighed for some shade from the scorching sun, and there were fears lest the heat should prove too much for any of the patients. Happily, none had to fall out, and at 3.30 we heard the distant sound of cheering and the voices of the Wynburg school children singing the National Anthem. Then came the welcome clatter of horses' hoofs, and up the hill cantered the advance guard of the Royal escort, consisting of a detachment of the "Peninsula Horse," followed by the Lancers. These drew rein just opposite our men as the Duke and Duchess alighted from their carriage, part of the way down the hill, at No. 1 General Hospital, and then slowly walked up the road to No. 5 General Hospital. As they approached, all our patients stood "to attention" with the exception of those who were not well enough to rise, and the Royal salute was sounded on the bugle, while a detachment of the R.A.M.C. Base Details presented arms. The commanding officers and superintending sister were presented to the

Duke and Duchess, who passed leisurely through the lines of men conversing with the medical staff on the hospital and its arrangements, until they reached the boundary of the camp where they re-entered their carriage and drove off to finish their tour through the suburbs of Capetown. Immediately the order was given to "fall out," many of the patients ran to the end of the camp which overlooked the road along which the Royal carriage had to pass. They returned greatly elated, saying that they had had a bow from the Duchess all to themselves, and that "she did not even look at the civilians who were cheering lustily the opposite side of the road." I may add that the soldiers considered themselves greatly honoured by the Duke and Duchess walking through the camp instead of remaining in their carriage.

### Next Day's Entertainment.

But the festivities in connection with the Royal visit were not yet over, for Thursday was to be devoted to the entertainment of the patients. All the morning the sisters and staff were busy preparing for the patients' tea, and very pretty the tables looked when they were finished, with their great jars of arum lilies—which would have cost a small fortune at Covent Garden—with piles of cakes and tarts and most appetising bread and butter. The rain, which had been threatening all day, kept off, and at 2.30 commenced the first part of the entertainment, a cricket match between the R.A.M.C. of No. 5 Hospital, and the R.A.M.C. Base Details. The contest was keenly watched by the patients and visitors, and great satisfaction was expressed when our hospital proved the victors. The only disappointment we had was that the band of the C.G.A., which we hoped would have played to us during the afternoon, was prevented from attending owing to a previous engagement in Capetown.

### The Chief Feature.

At five o'clock the tea-bell rang, and then began the chief feature of the day's performances. Each patient found before him a large plate of ham and tongue and pickles, to which he devoted his attention before attacking the heaped-up dishes of cakes, etc. It was quite sufficient to see the happy faces of the men, without listening to their complimentary remarks, to know that our efforts of the morning were fully appreciated. I think that the only anxious person in the room was the sister of the enteric ward, as she watched her convalescents helping themselves to pickles, and great was her relief the next day to find their temperature still normal. Some of the patients had their tea carried to them in bed; only one was allowed no deviation from his milk diet, and he has been promised a cake to himself when he is well enough to eat it. At the close of the tea each man received a cigar, and they had previously been presented with a handsome pipe as a memento of the day. The cost of the entertainment had been defrayed through the generosity of the Royal Reception Committee, and while the tea was in progress the men accorded a hearty vote of thanks to the committee, which was at once, at their request, forwarded to the Mayor by their commanding officer through the telephone.

### Tommy and the Sister.

It was six o'clock by the time the tea was over, and by seven the patients had all met again in the recreation-room for a "sing-song" which lasted until 9 P.M., when, after singing "God Save the King," they dispersed to their respective wards. The opinion of the entertainment is best expressed in a remark made by one of the Tommies the following morning: "A few teas like that, sister, would soon fatten us up. It was just grand, and we couldn't sleep all night for talking about it." But the sister was inclined to express her view of the cause of their sleeplessness by the one word—"pickles."

### The Nurses' Bookshelf.

A Woman's Memories of the War. By Violet Brooke-Hunt. (London: James Nisbet and Co. 1901. Price 5s.)

Among the many books about South Africa which we have met with this strikes us as being one of the healthiest and most satisfactory. Although the writer found herself much in touch with nurses and hospitals and medical officers, she did not go out as a nurse, but rather because she had been much interested in young men, and young men's institutes. She had been led to establish reading rooms and young men's institutes at and about her own home in Gloucestershire, and the feeling had been impressed upon her that wherever young men were to be found in any number a need existed for some form of institute where bodily comforts and harmless pleasures could always be found, while at the same time all those influences which made for righteousness would never be lost sight of. A move to London had shown her that a young trooper in the Blues is really very little different from his civilian brother, except that he is more ready to grasp the club spirit, to appreciate it to the full, and to accept its discipline as a matter of course; and when the war came there came also a strong feeling that she ought to go to help in some way or another to soften what must be a hard life for these splendid soldiers of ours. So she went and this is the history of her doings. For this history and all the interests which it opened up, and all the good that the writer was able to do, and about which she speaks in so simple a fashion, we must refer to the book itself, but we cannot omit to say that while the story is told in a charming and interesting style which carries one on without a break through all her wanderings, simple, homely, and even commonplace as were many of the things she did, there is an undercurrent of common sense and of readiness to make the best of everything, and more than that there is a power of penetrating the deeper side of men's natures, which makes the whole book extremely pleasant reading to those who have faith in our "common" soldiers. What one may call the "travel" part of the story, the journeyings by South African trains, the halts, and the adventures, all these may be read in other books, although perhaps not so well told as here; but what is really of interest is the account of what was done for the men, and the way all worked together to secure the success of soup kitchens and convalescent camps and soldiers' institutes, and especially the touching insight that is given in regard to the religious feelings which permeated these rough soldiers and which under stress of sickness or from other causes they occasionally laid bare to one who evidently was regarded by them as a trusty friend. It is a very nice book, the reading of which will, we are sure, give pleasure to the mothers and sisters of many a good soldier in South Africa.

### "The Hospital" Convalescent Fund.

The hon. sec. acknowledges with many thanks the receipt of 5s. towards the above fund from M. A. Barnes Groom.

### To Nurses.

We invite contributions from any of our readers, and shall be glad to pay for "Notes on News from the Nursing World," or for articles describing nursing experiences, or dealing with any nursing question from an original point of view. The minimum payment for contributions is 5s., but we welcome interesting contributions of a column, or a page, in length. It may be added that notices of entertainments, presentations, and deaths are not paid for, but, of course, we are always glad to receive them. All rejected manuscripts are returned in due course, and all payments for manuscripts used are made as early as possible after the beginning of each quarter.

### The Nursing of Tropical Dysentery.

By a Sister in a Colonial Hospital.

To some of our sisters whom the war has called to South Africa, the nursing of dysentery cases may be proving a somewhat new experience. It will at least form an instructive and a useful succedaneum to the English training of those who have not been abroad before. It has occurred to me that some notes on this disease, by a sister who received her hospital training in a tropical country where dysentery is endemic, may prove of some interest for purposes of comparison with the features it is exhibiting in the military hospitals at the seat of war.

### European Cases in the Tropics.

I must premise my remarks by stating that dysentery cases usually present some distinction of type depending upon the race to which the patient belongs. My own cases for the most part have been Europeans, Indians (N.W.P.), Fijians, and Melanesians, and therefore what I can say about the subject must be understood as applying specially to those peoples. In the tropics, I am afraid that the European is more often overtaken by the acute form of the disease than the native; at least that form which is acute from its earliest stage. When the native suffers from acute dysentery it is usually because, either from ignorance or from neglect, he has not come under medical treatment at the beginning, or because such native treatment as he has submitted to has been crude and unscientific, empirical, or founded on witchcraft. The key by which to recognise the type and tendency of a case of dysentery is inspection of the stools; this is a function of the nurse as well as of the physician, and should never be neglected nor left unrecorded in writing. There will be other symptoms as well for her observance and report; temperature, pulse, pains, tongue, condition of skin, quality and amount of sleep, prostration, and so forth—not forgetting a careful notice of the manner in which the drugs prescribed by the doctor are borne. Fortunately, headache rarely or almost never occurs during the progress of a case of dysentery, so the patient is spared that trouble and distress. And well he may be, for the severe and frequent tenesmus, the ever recurrent rectal bearing down and involuntary straining at stool, the exhaustion incident to the loss of blood and enforced spareness of the diet, together with the nausea and other worries of the ipecacuanha treatment, afford an ample amount of woe for him to put up with.

### A Serious Scourge.

In the earlier years of settlement in a new country, especially if a tropical one, dysentery is often a serious scourge. The exposure of miasmatic swamps by felling the bush, and the turning up of previously uncultivated soil, the indifferent food and impure water available for domestic needs, exposure of the body to the solar rays, to rain or to night chills, or to excessive physical labour, and its evil consequences in the way of bad alcoholic liquors and too much of them, all tend either as exciting, or predisposing, causes as the case may be, to claim the pioneer colonist, the soldier, or the foreign coolie, as a victim of a dysenteric attack. Not infrequently it appears in a distinctly infectious form, and so gives rise to epidemics of a more or less circumscribed character. There is no limit to the age at which patients may be attacked. I have seen it in infants of six months and in people of fifty, and females as well as males suffer from it, though not to such a great extent, as they are not so much exposed to the causes already enumerated. All the native races may suffer, and Europeans frequently have it very severely. Among the latter it is often preceded by constipation, but in the acute cases the onset is sudden; there is perhaps a rise of temperature, severe abdominal pain, tenesmus, thirst, furred tongue, exhaustion, and frequent evacuations containing blood and mucus mixed with fæcal matter; and very often large quantities of blood and mucus alone. I have noticed that it is chiefly in European patients that pyrexia occurs; with natives it is rather the rule to have a normal and subnormal temperature. The pulse varies as the case progresses; it has no very distinguishing features, but is usually weak and accelerated. The medicinal treatment may vary according to the race and age of the patient, the stage of the disease, and the experience of the doctor; but rest and diet are always two very essential points.

### The Two Treatments.

Two methods are recognised, however, as pre-eminently orthodox; the ipecacuanha treatment and the saline treatment. The former has two drawbacks; it is apt to provoke troublesome nausea, and the drug, whose keeping qualities are none of the best, is of no avail unless fresh and genuine. It is also somewhat expensive, and very nasty to swallow. Salines, on the other hand, keep indefinitely, and are cheap. They, too, are nauseous unluckily. In my own wards the pecacuanha treatment has always been the routine; the saline treatment has been tried but has not proved a favourite. One of our surgeons, who lately visited Singapore, told me that in the General Civil Hospital there the converse was the case, and that among Chinese and Malays a saturated solution of sulphate of soda or sulphate of magnesia, with a few drops of aromatic sulphuric acid, in cinnamon water, given hourly, until the stools ceased to be frothy or to contain blood, gave excellent results. In Fiji, if I may venture an opinion, I should say that the ipecacuanha treatment answers as well as one can wish; and it has held its own for 20 years. Most cases do well on half a dram of the powdered root, every night and morning, given alone, and followed by a single mouthful of water to wash it down; but if there is much vomiting it is preceded by Tr. Opii, xx m., and a mustard plaster over the epigastrium. This is a great help; and by its means the powder can often be retained, even when the stomach is sensitive. After a few days of this treatment there should be no blood in the motions; there may be mucus for a time, but in a fortnight the patient is usually well. Milk is the only nourishment allowed; five pints daily, and a little stimulant when necessary. Washing out the bowel daily with a solution of boric acid, 10 grs. to 1 oz. of warm water, or sometimes a saturated solution, often has a good effect. As soon as the inflammatory stage is passed, and the motions become free from blood and mucus, and are only liquid in character, the ipecac. is stopped; an astringent mixture is then given, and a light diet consisting of any farinaceous food, with milk and chicken broth, is allowed.

### Chronic Cases.

The most trying cases to doctor, nurse, and patient are those of chronic dysentery. They are met with chiefly in alcoholic Europeans and the older natives. The patient does not feel ill enough to stay in bed, and consequently retards recovery by going about. Of course in hospital they are obliged to keep still, but many do not come in until they are very emaciated and weak. In this condition the motions do not contain much blood, and often none at all, but large quantities of mucus; and there is much abdominal tenderness. The diet is restricted as in acute dysentery to milk, with perhaps an egg flip, sago and toast added. The chief feature of the medicinal treatment is a careful washing out of the large intestine daily with a boric solution, or with nitrate of silver $\frac{1}{2}$ gr. to an ounce of water, or sulphate of zinc 2 grs. to an ounce. I have observed large quantities of mucus passed after an injection of nitrate of silver, 2 grs. to an ounce with 2 m. of Tr. Opii., preceded by a simple douche. One of the best remedies is a change to a more temperate climate, but I have known the disease to recur in several instances after the patient has returned to the tropics. In one case of acute dysentery I have seen the patient lose daily quite 8 ozs. of blood for nearly a week, during which time he was almost in a state of collapse.

*(To be continued.)*

### Echoes from the Outside World.

### AN OPEN LETTER TO A HOSPITAL NURSE.

I LITTLE thought, when I alluded so hopefully last week to the reports of the progress made by President McKinley, that in a few days his life upon earth would be ended. It is still difficult to realise that the optimistic bulletins of the early part of the week could have been the true opinions of the eminent men who, within a short time, in spite of all their efforts, were unable to save the patient. Some of the telegrams received privately from America have given rise to an idea that it was known by the surgeons from the outset that the wound would prove fatal, and that the expressions of hope—I had almost written of certainty—which characterised their published utterances were due to pressure from the Stock Exchange, where the only chance of avoiding ruin to thousands was to let the bad news transpire after an interval. It is hardly possible to believe that any exigencies of business would so influence medical men that they would buoy up, not only the patient himself, but his family, with what they knew to be fallacious expectations. The cause of death, it is now stated, was gangrene, not only around each wound, but all along the bullet track, and some maintain that the bullet was, so as to make assurance doubly sure, poisoned by the assassin. In any case, the police have shown their wisdom in removing Czolgosz elsewhere to avoid more and fiercer attacks upon the prison. To ensure his safety he had to be dressed up in a constable's helmet and coat. He is to be tried at once, as, unfortunately, there is no longer a reason for delay, though it is too much to anticipate that the expiation of his crime will have any real effect upon the other madmen who call themselves Anarchists.

THE glimpses of the sick room which have come to us now and then through the medium of the press have done much to reveal the quiet faith and the simple nature of William McKinley. When he submitted to the anæsthetic for the operation directly after the attack, as he drifted off into unconsciousness he was heard to murmur the Lord's Prayer; at the final interview with his wife, when he did his best to comfort her, his parting sentence was, "God's will, not ours, be done;" and the last words he was able to speak before he sank into the comatose condition in which he died were "Nearer, my God, to Thee." All our hearts of necessity go out in womanly sympathy to poor Mrs. McKinley, who has now the burden of ill-health to bear with no strong loving presence near to help her. For many years of the President's married life he had been a tender nurse as well as a devoted lover to his delicate wife, and probably her daily prayer is that she may, ere long, be permitted to follow him. Though they had been wedded some years, they had never grown beyond the days of courtship it is said, and were as much in love with each other in 1901 as in 1870, when Miss Ida Saxton, the daughter of a banker in the little town of Canton—who, though her parents were in a good position, was placed by her father as a clerk in his bank—attended to a young attorney, and in receiving his business instructions, found she had also gained his heart. Having obtained her consent, the young man sought an interview with Mr. Saxton, and in reply to the curt questions "Who are you, and what do you want?" replied simply, "My name is William McKinley, and I want your daughter in marriage." The two little girls who were born to the President and his wife both died before they were five years of age, so that the husband and wife had literally been all in all to each other for many years.

MR. THEODORE ROOSEVELT—the name is honoured in the United States—who becomes President for the unexpired years of Mr. McKinley's term of office, is a man of a very different stamp to his predecessor. By birth he belongs to the aristocracy of America, he is broad and square-shouldered, extremely muscular, and stands 5 feet 9 in his stockings. Without being actually handsome, his appearance is very distinguished, and he is almost universally known as "Teddy," in the same way as Mr. Chamberlain is popularly designated "Joe." The man in the street, as well as the woman in society, applies this sobriquet to him in the same unconscious manner. It is said that those who do not agree with Mr. Roosevelt's political views admit that they can forgive him much for his absolute honesty, but many aver that he is at present something of a "dark horse," and the characteristic remark of a compatriot is worth repeating as giving the view held about him. "I'm thinking that when Teddy's done with America it will, may be, require another Christopher Columbus to find what's left of it." Unlike most men he is a great huntsman as well as a great reader. He was educated at Harvard University. Plutarch is his constant pocket companion, and he has written several books which attest his versatility. He spent some of his early years amongst the Cowboys of the Far West, where he was adored for his indomitable courage, and when the Hispano-American war broke out he threw up his office, raised a corps of rough riders, of whom he became colonel, and went to Cuba. He is married, and has several children. He often stays in London, and though he has not a drop of English blood in his veins feels, he says, more at home here than on the Continent. An excellent impression has been created by his manner and words on his assumption of office.

THOSE who remember that it was entirely owing to Queen Alexandra that the lamps for the cure of lupus were started at the London Hospital, will not be surprised that one of the afternoons during the Royal visit to Denmark was devoted to a long visit to the new light cure hospital at Rosenvaenget. The party consisted of the King and Queen of England, the Dowager-Empress of Russia, the Crown Prince, and the Grand Duke Peter of Oldenburg. They were received at the hospital by the Professor, who showed them all his latest inventions, and several lupus patients had the honour of a talk with the Royal visitors. Over an hour was spent in the institution, and when they departed the Professor expressed himself as much surprised that His Majesty showed such an extraordinary knowledge of the details of the process. But it is a subject in which the King, as well as the Queen, takes the deepest interest; and judging by his speech at Marlborough House recently on the subject of cancer and consumption, he, in common with many others, is hopeful that the action of light may some day be able to do something to mitigate also those two scourges which at present claim yearly so large a number of victims.

THE behaviour of the Duke of Orleans a year or so before the death of Queen Victoria in showing his appreciation of some disgraceful caricatures of Her Majesty, greatly disgusted the people of England, and most of us tried to forget that such a person ever existed. Accordingly, when amongst the donors to the memorial to Queen Victoria, the name of the Duke of Orleans appeared, public surprise was expressed that any contribution should have been accepted from one who went out of his way to offer her an insult while she was alive. This sentiment found vent in some of the newspapers, but an authoritative statement has now come from Copenhagen, explaining why the money has not been returned. It points out that the severe comments following the offering were evidently based upon misapprehension, and that the writers could not have been aware that the Duke wrote a letter to Queen Victoria, apologising and begging her pardon. The late Queen's answer, it was added, was conceived in the most generous spirit, Her Majesty forgiving him in her own name and in that of her Royal Family. This explanation will be warmly welcomed by John Bull and his womenkind; and, in justice to the headstrong young Prince, who, though he behaved foolishly, was man enough to admit it, deserves all the publicity which, I am sure, the gracious lady who so freely forgave the offence would have wished it to receive.

### A Book and its Story.

### NURSING THE BOERS.*

MADAME BRON'S "Diary of a Nurse in South Africa," translated for English readers by G. A. Raper, will be read with special interest, not only by her sisters in England to whom it must necessarily appeal forcibly, but also by everyone who is in sympathy with personal records of dangers undergone, and privations heroically endured "for the work's sake." The diary is "a series of impressions written on the spur of the moment." It has no literary pretensions, but the reader is carried along by the simple vivid force which characterises it. Written under circumstances that left no space for fine finishing, these daily vignettes are dashed off with Rembrandt-like effect. Deep shadows predominate, and lurid lights flash on scenes which, in spite of their weird intensity, are not without saving gleams of pathos and humour.

In a biographical note affixed to the diary, we are told that Mdme. Bron, from her earliest years, has devoted herself to works of charity. The sick and suffering have ever had her active sympathy. As a mere child, she and a brother, joined the staff of a private hospital in Brussels, opened for the reception of the wounded in the Franco-German war. After her marriage with a wealthy manufacturer, M. Arthur Bron, she undertook the establishment of schools and hospitals for his workpeople, and nursed them when sick in their own homes. During the riots which occurred in the Charleroi district some fifteen years ago, so great was her personal influence over the people that, although looting and burning of factories was going on in the neighbourhood on all sides, Mdme. Bron calmly approached the excited mob, and so soothed them by her magnetic persuasiveness that they retired without injuring any of her husband's property. Her sympathy with the poor and oppressed led her also to identify herself with Socialistic movements, but she withdrew from the Belgian working-class Socialist party which she had joined in her ardour for the amelioration of their condition, finding its aims and basis neither wide nor deep enough for her generous catholic mind. In another sphere also Mdme. Bron has had regretfully to forego the high ideal she had formed of an oppressed people fighting for freedom, and in consequence she has resigned her place on the staff of the Dutch and Belgian ambulance formed by the United Red Cross Associations. She is now in the service of England as a nursing sister. In the summer of 1900 she was recalled by a telegram announcing the sudden death of M. Bron. Returning to the Cape as soon as circumstances permitted she took up her position which she retains at the present time. A portrait of her on the opening page of the Diary in uniform, not an English one, and taken probably when she first went out to South Africa, shows that of a woman of much force of character. The face is dark with strongly-marked eyebrows and a powerful chin. The lips show self-command and the nose the power to direct and control others. The diary begins when on duty in Jacobsdal early in February, 1900. She writes, standing at the door of the improvised hospital, until now a Kaffir church: "Sixty Boers with a maxim have just gone past about 50 yards away from the hospital, all clustered together on horseback; with their big hats and unkempt beards, they make fine figures. They are extraordinary horsemen, riding barebacked at eight years of age, and developing into so many chase-loving centaurs. . . . Away the group filed in the fading daylight under a sky swollen with storms, and gradually they melted away into the great dusky desert that spreads out on every side. Dimly I saw them jump off at the water to let their horses drink. As they started again they struck up a very slow, soft, and infinitely sad chant, very grand and captivating in such surroundings."

The writing of her diary was a matter of difficulty to "Sister Alice" at times, and was generally done at odd moments on night duty. On one occasion, she says, "I have everything on my hands now, one of the nurses having been discharged while the other is struggling with incipient typhus, so that I am practically on duty 12 hours at a stretch, with hardly time to sit down; but the patients are doing better, so I am able to jot down my notes." The Boers thought nothing of a woman being on duty for 12 hours, but that she should appropriate spare moments for writing, appeared to them a task beyond the strength of woman! Writing in full view of her sick and wounded Boer patients they say to her, "Zuster, you'll make yourself ill if you write so much." "I look at them in amazement. I jot down these incoherent notes whenever I had a few comparatively restful moments. My mind remains as active as a stationmaster's in holiday time, when the problem of running several trains on the same line has to be solved." Mdme. Bron suffered keenly from the incapacity and cowardice of the two Boer attendants who were forced upon her. She much preferred the Kaffir hospital servants. "These Boer attendants were men who had simply put on the Red Cross badge to escape from active service. They do not know even the A B C of sick nursing. . . . I dispense with their services as much as possible, and look after them very closely; but their children and grandchildren will probably speak with bated breath of their services, and when a few more generations of humbugs have had their say, these attendants of mine will doubtless figure in history as so many celestial beings descended from heaven to succour and console a band of heroes on earth." Before the bombardment of Jacobsdal a lively discussion was going on in the little hospital between the sister and her patients. A few hours earlier some Boers had come in with the intelligence, "That after all the English seemed to be moving on Bloemfontein." "Ever since I came out I had heard the Boers extolling their own courage and saying the English were afraid. I invariably prefer to form my own opinion, to which end I ask a great many questions. 'Why don't the English come on?' 'Because they are afraid.' This struck me as strange, but I was then in the first flush of my admiration for the Boers. My enthusiastic imagination adorned them with all the virtues it had fattened on in Europe. The veil of illusion was very soon torn from my eyes, and I saw clearly. . . . Later I tried to collect the scattered fragments of my enthusiasm and to struggle against the rude shattering of my beliefs, but the reality has never failed to rise up implacably and exclaim, 'O blind, will you not see? O deaf, will you not hear?'" During the bombardment of Jacobsdal she writes: "The English are coming along with their guns. I must go and get something to eat, otherwise I may have to lunch off the fragments of a shell." Whilst at her hasty meal a single cannon shot was fired, and although, before leaving, her Boer attendants had indignantly protested in reply to her inquiry, "Will you be afraid? of which I had my doubts," yet when she rushed back to the hospital two panting, terrified figures were encountered, wild with fright; one had lost his hat. "These were my two attendants!" Later, when some British soldiers came in to hoist a white flag in place of the Orange Free State colours, until then flying over the hospital roof with the red cross above them, the officer with them inquired, "Were you not afraid, Sister?" "I looked him straight in the face and replied, 'A good sister cannot be afraid—she is a soldier.'" From the privations and hardships, bordering on starvation on one occasion, Mdme. Bron's health suffered severely, but nothing could daunt her indefatigable spirit, and the whole narrative is one of stirring interest from first page to last. It is not merely a diary of incidents, it is one of character also.

* Diary of a Nurse in South Africa. By Alice Bron. Publishers; Chapman and Hall. Translated.

### Everybody's Opinion.

### TRAINING AND NURSING IN POOR LAW INFIRMARIES.

"SCOTSWOMAN" writes: I have been greatly interested by your report of the deputation of the Workhouse Infirmary Association to the President of the Local Government Board, and I should be glad to hear whether anything is being done on similar lines in Scotland, where for long we have boasted of leading the van in matters relating to Poor Law administration. I have for 20 years been a constant visitor at a small poor house—a poor house too small to claim the trained nurse whose employment is "suggested" by the Scottish Local Government Board, and where the so-called "nursing" is done entirely by paupers, very frequently by imbecile paupers. During that time I have seen severe cases of cancer, bronchitis, etc., and constant cases of consumption, sores of various kinds, heart disease, epilepsy, paralysis, and so on. Many of these cases must have required constant attention, not to speak of night nursing; and the attention given by a fellow pauper may be better imagined than described. Besides the above there are, of course, constant midwifery cases, and the medical officer, I may mention, resides a considerable distance off, and is a busy country doctor. Now, has the time not come for an order, similar to that recently issued in Ireland, to be made by the Local Government Board of Scotland, which shall enforce the appointment of trained nursing, and establish a standard of nursing which will prevent inefficient women being appointed to perform the duties devolving upon them? We should avoid falling into the errors which have been made on the other side of the border, by establishing a sub-department of the Local Government Board to deal with the whole subject of nursing in poor houses, and if necessary undertake the training of its probationers in properly equipped schools. The treatment of our sick and aged paupers is fast becoming a disgrace in an age of civilisation, and in the eyes of those nations which have devised other and better methods of meeting the evils of pauperism.

### LADY HARBERTON AND RATIONAL DRESS.

"S. L. C." writes: In your issue for September 7th Viscountess Harberton has an article on "A Fashion Producing Disease," in which I was very much interested. If I held the social position of the writer I should not hesitate as to wearing "rational dress." As it is, I very often feel inclined to do so, although I am "only a district nurse (Queen's)," especially when I am a witness of some of these trails "which are nothing but a nuisance to the wearer and to the community at large." One evening in the last week a lady called upon me, and after she left I found on the carpet of my sitting-room a little bunch of tobacco, a dead beetle, and two used matches. At first I could not make out how such things got there, but after a moment's consideration I came to the conclusion that my visitor must have brought them in her trail, "which was quite three-quarters of a yard on the floor." "L. T." suggests as an improvement buttoning the trailing skirt to the waist when out walking. I am afraid that "L. T.'s" suggestion will not be readily taken up, as I generally see people in these kind of dresses when they are out of doors. Therefore, I assume that the wearers wish all their "friends, acquaintances, etc." to be aware of the fact that they are not behind with the fashions however ridiculous they may be. Although I agree with Lady Harberton in most things which she has written, I must say that her knowledge of the nurse's uniform is not very extensive. I have been a nurse for four years, and during the whole of that time I have only seen one trained nurse who wore her dresses trailing; in fact, most of the institutions that I know prohibit "extra-long skirts" for their nurses, either as indoor or outdoor uniform. As to the headgear with streamers attached, nurses are not to be blamed for that fashion. If they belong to certain institutions they have to wear them whether they like them or not, also the clumsy and inconvenient cloaks. The sketch, "An Example of Rational Dress," is certainly not extra-becoming and rather too much like the male attire, especially as the bicycle by which the figures stand is a man's bike. For my own part, the sooner the rational dress comes into fashion the better pleased I shall be, as I spend half of my days on the bicycle.

### MONTHLY NURSES.

"NURSE MARY" writes: "Mother's" letter contains an element of truth and common sense, but its general tone is undoubtedly a reflection on the monthly training of nurses. Judging from her letter, she must have met with an exceptionally incompetent nurse. As a rule, nurses who have received a hospital training, and in addition a monthly certificate for nursing, have spent part of their general training in the children's wards, and have had charge of little ones, whose ages range from a few weeks to several months, all artificially fed, principally on cows' milk; so that this experience should enable them to do private nursing. Surely "Mother" does not include the latter class in her category of incompetent nurses. As a monthly nurse of some years' standing, I must confess that the milk nature supplies is not always, either in quantity or quality, to be preferred. I have nursed cases where sterilised cows' milk, to which is added thin barley water, properly prepared, proved far more nutritious and beneficial than even the mother's milk. Flatulence, hiccough, a restless sleep, indigestion—evidenced by child's unhealthy motions—are often the result of natural feeding, whilst I have known a mixture of cows' milk and barley water to conduce to a sound, healthy sleep. Mothers are often very ignorant of the requirements of their own children. Some mothers make it a point to lift their child from the cot or bed each time it cries, and of putting it to the breast instantly, believing this to be a panacea for every ailment; consequently the poor child, whose stomach is full to overflowing, suffers from indigestion, and the nurse's work is to minister to, and soothe the little mite until sleep, "Nature's restorer," comes to its aid. A well-trained, careful and conscientious nurse will know how to clean bottles and prepare infants' food, also the quantity of food necessary for them. If nurses would do their work in a less mechanical manner, and bring both knowledge and common sense to bear, we should have fewer complaints, and they themselves would find these helpless little ones, so dependent on them for every comfort, far more interesting patients than even the mothers themselves.

### THE HOURS OF NURSES.

"A NURSE'S MOTHER" writes: I have read carefully and with much interest an article in your last issue, "The Case of the Shop Assistant." Without wishing in any way to take exception to what is said, it struck me very forcibly, especially after the testimony of 300 medical men, that the protest against long hours should go further and include our hospital and private nurses. There are few professions that are so arduous or so exhausting. The hours of work are longer than those of the shop girl or the ordinary factory hands, yet I have seen no plea on behalf of our probationers and nurses. Anyone acquainted with hospital training knows that numbers of ladies who wish to enter this most honourable profession break down under the strain of the first month's work. These are not young girls, for the rules of the larger hospitals preclude the entrance of a probationer under 24 years of age. The change from home life and carpeted floors to the long hours of standing or walking on wooden ones is often agonising to the unaccustomed feet, but the rule of the hospital is inexorable and the weary hours drag on without relief to the suffering nurse. Even when this stage is passed the long hours are a terrible strain. Take the case of the probationer on day duty. Speaking generally, the hours are from 7 A.M. to 9 P.M., with two hours off duty daily or four on alternate days. I know of only one London hospital giving four hours daily. This gives an average per day of 12 hours, less the hurried meals, which can hardly be construed into rest, and 12 hours per day for seven days mean 84 hours per week (less, of course, the short time for meals and a day off during the month). How does this compare with the working man's week of 52 hours or of his ideal week of 48? Then the nurse on night duty. Her hours are from 9 P.M. to 9 A.M., with a meal in the ward during the night. Here are the full 84 hours per week in the ward. As I have said, many probationers do not get beyond their first month's trial, and many cannot bear the strain more than six. Yet it may easily be imagined that the qualities of mind and disposition that would make a superior nurse are not always associated with the physical robustness needful for the present system, and thus many valuable nurses are lost to the profession. The limited area of the hospital often results in limited area in the nurses' sleeping apartments, an additional reason for more hours in the open air; but when the time for rest and recreation arrives the nurse is often too tired to make the best use of it. Two hours from leaving the ward to returning to it, give short time for change of scene and thought, and a walk in the streets of London is hardly a remedy for exhaustion, yet the very nature of a nurse's duties require she should be at her best at all times, and never the victim of overwork. There is a sort of grim irony in the fact that in the very places designed for the establishing of health, under the very eyes of our physicians, the conditions are present which almost inevitably induce physical deterioration. After a few months' attendance in the temple of health the vestal emerges, not bearing about her the spirit of the place, but pale and distraught. The private nurse's position and duties are on different lines, and depend on the nature of the "case," the help she receives, and the thoughtfulness and consideration of the family. In some instances, where the duties are light and the nurse treated kindly, the position leaves little to be desired; but in very many the nature of the complaint is trying and exhaustive, and the nurse is expected to take entire charge, sleeping in the patient's room. It often happens that though there is ample need for a second nurse, economic reasons prevent, and thus double duty is inevitable for the one. I have a letter from a nurse of long and varied experience who says: "I have often been on duty 140 hours in the week." The remedy here can only arise from a fuller recognition of what is due to a nurse from her employers. But in the case of the hospitals, where the plea of the authorities is always lack of means, I would venture to suggest that in addition to the splendid charity that sustains our hospitals, some well-organised scheme might be devised whereby the convalescent patient might contribute some portion of the expense his sojourn has caused. Many of our artisans and working men would, I feel sure, gladly fall in with such a scheme, and it is possible that under such a system the point could be reached that three times eight hours would fill up the twenty-four of the day with three sets of nurses.

### Appointments.

Broad Clyst Nursing Association.—Miss Tobin has been appointed district nurse. She was trained at Victoria House, 46 Gillingham Street, Leicester Square, London, and has worked for the Beds. Rural Nursing Association for three years.

Chester Isolation Hospital.—Miss Sarah Martha Edwards has been appointed charge nurse. She was trained at Salop Infirmary for three years, and has since been staff nurse at the Royal Chest Hospital, City Road, London; charge nurse at the North Western Fever Hospital, London, and the City Hospital Sheffield; and private nurse at Cardiff.

City Hospital, Park Hill, Liverpool.—Miss Jennie Brooke has been appointed charge nurse. She was trained at the Royal Infirmary, Sheffield, and St. Helen's Sanatorium.

Colonial Hospital, Gibraltar.—Miss Margaret Farrow has been appointed sister. She was trained at the Royal Albert Edward Infirmary, Wigan, and has since been engaged in private nursing, and as staff nurse at the Rawcliffe Hospital, Chorley.

Reigate Union Infirmary.—Miss Mary Teresa Guthrie has been appointed superintendent nurse. She was trained at Whitechapel Infirmary, where she has since been staff nurse. She holds the L.O.S. certificate.

South Devon Hospital, Plymouth.—Miss Mildred Jackson has been appointed night sister. She was trained for three years at the Royal Infirmary, Liverpool, where she has since served on the private nursing staff, and as temporary sister.

Spalding Union Infirmary.—Miss E. L. Allen has been appointed nurse. She was trained at Pontefract Union Infirmary.

### Croydon Infirmary.

### OFFICIAL ANNOUNCEMENT OF TEN RESIGNATIONS OF NURSES.

The Croydon Board of Guardians met on Tuesday for the first time after the summer recess. The Infirmary Committee reported as follows:—

That Agnes Gordon, assistant nurse of the maternity wards, and Probationer Nurse J. Grey left the Infirmary on August 20th, as the result of the action of the Visitors on that date.

The Committee had received letters from the undermentioned probationer Nurses resigning their appointments, namely:—Edith M. Gooch, August 14; Fanny Shird, August 14th; Kathleen Pomeroy, September 6th; Helena Mathieson, September 8th; Marie Feetham, September 8th; May Willoughby, September 8th; and Annie E. Marsh, September 9th.

The Committee recommended that the resignations be accepted, and gave directions for the nurses in question to be allowed to relinquish their duties at the expiration of one month respectively from the above-mentioned dates.

The Committee authorised the medical superintendent to at once take steps to fill up the vacancies caused by the resignation of probationer nurses, and recommended that, in the event of an emergency arising, the medical superintendent be allowed a free hand to engage temporary nurses to fill the vacancies.

The Committee desired to point out to the Board that the acceptance as a matter of course of the resignations of probationer nurses was somewhat irregular, inasmuch as probationer nurses when appointed signed an agreement to serve for a period of three years from the date of their appointment. They did not, however, suggest that officers should be retained who were dissatisfied with the conditions under which their services were to be given, but merely suggested that it should be notified to probationer nurses generally, through the medical officer, that these officers were not entitled to leave at any time they might choose to resign, but that their resignation was conditional on the Board being disposed to release them from their engagement.

Dr. McClintock, assistant medical superintendent, reported that Clara A. Curd, charge nurse of the lying-in ward, had forwarded to him her resignation, dated September 5th. The Committee recommended that Nurse Curd's resignation be accepted as from October 5th, and that the clerk be instructed to advertise for a successor to Nurse Curd on the usual terms.

The Committee considered the cases of the late officers, Agnes Gordon (assistant nurse in the lying-in ward) and Jenny Grey (probationer nurse), and they recommended that Miss Gordon be paid her salary and a sum equal to the value of her emoluments up to the 27th inst., and that Miss Gray be also allowed the value of her emoluments up to the date to which her salary had been paid to her, viz., the 14th inst.

Mr. Densham, Chairman of the Committee, moved the adoption of the report, but made no reference to the resignations.

Mr. G. J. Shirley said he had seen it stated in "*The Hospital*" *Nursing Mirror* that 13 or 14 of their nurses had resigned recently. The report stated seven or eight had resigned, and perhaps the Chairman would explain why they had lost so many.

Mr. Densham appealed to Mr. Shirley to leave this matter over till later in the day at the close of public business. This was agreed to, and the report passed.

### For Reading to the Sick.

"THE JOY OF DOING KINDNESSES."

SINCE service is the highest lot,
And all are in one Body bound,
In all the world the place is not,
Which may not with this bliss be crowned.

The sufferer on the bed of pain
Need not be laid aside from this,
But for each kindness gives again
"The joy of doing kindnesses."

*Mrs. R. Charles.*

Who hath aught to love, and loves aright,
Will never in the darkest strait despair;
For out of Love exhales a living light,
A light that speaks—a light whose breath is Prayer.

*H. Coleridge.*

*One* only knows. Yet if the fret
Of thy weak heart, in deep regret
Needs a more tender comfort yet;

Then thou may'st take thy loneliest fears,
The bitterest drops of all thy tears,
The dreariest hours of all thy years;

And through the anguish there outspread,
May ask that GOD's great Love would shed
Blessings on one beloved head!

*A. Procter.*

There is no beautifier of complexion, or form, or behaviour, like the wish to scatter joy and not pain around us.

*R. W. Emerson.*

We may, if we choose, make the worst of one another. Every one has his weak points; every one has his faults; we may make the worst of these; we may fix our attention constantly upon these. But we may also make the best of one another. We may forgive, even as we hope to be forgiven. We may put ourselves in the place of others, and ask what we should wish to be done to us, and thought of us, were we in their place. By loving whatever is lovable in those around us, love will flow back from them to us, and life will become a pleasure instead of a pain; and earth will become like heaven; and we shall become not unworthy followers of Him whose name is Love.—*A. P. Stanley.*

I believe where the love of God is verily perfected, and the true spirit of government watchfully attended to, a tenderness towards all creatures made subject to us will be experienced; and a care felt in us, that we do not lessen that sweetness of life in the animal creation, which the great Creator intends for them under our government. . . . To say we love God as unseen, and at the same time exercise cruelty toward the least creature moving by His life, or by life derived from Him, was a contradiction in itself.—*John Woolman.*

Not a prayer, not an act of faithfulness in your calling, not a self-denying or kind word or deed, done out of love for Himself; not a weariness or painfulness endured patiently: not a duty performed; not a temptation resisted; but it enlarges the whole soul for the endless capacity of the love of God.—*E. B. P.*

### Notes and Queries.

**The Editor is always willing to answer in this column, without any fee, all reasonable questions, as soon as possible.**
**But the following rules must be carefully observed:—**
**1. Every communication must be accompanied by the name and address of the writer.**
**2. The question must always bear upon nursing, directly or indirectly.**
**If an answer is required by letter a fee of half-a-crown must be enclosed with the note containing the inquiry.**

*London Needlework Guild.*

(231) I should be glad if you can give me the address of the London Needlework Guild, which makes grants of clothing to institutions.—*A.F.R.*

Write to the Honorary Secretary, Mrs. Basil Ellis, Ockshot, Surrey.

*Pau.*

(232) Can you tell me whether there is a nursing home, or English nurse at Pau, and also a masseuse.—*C. H.*

There is no nursing home, but we do not know whether any English nurse resides at Pau.

*Stove for Hospital.*

(233) Can you tell me the best kind of coal-burning stove for a room used as an operating theatre? The room is 15 feet by 14 feet, with a large skylight; and the stove will be the only means of boiling the water for operations as well as of warming the room. This is a cottage hospital, and it would save useless expense if no mistakes were made before the best kind of stove was secured for the purpose.

A good specimen of an ordinary kitchen stove with boiler, so arranged with circulating pipes and cylinder as to provide a good supply of hot water, would serve the purpose.

*For Probationers.*

(234) Would you kindly make it known that I take probationers from time to time who are too young for hospital training? I give them board and washing, but no salary.—*Matron.*

Your best plan is to advertise.

*Free Maternity Training.*

(235) I am a trained nurse, 35 years of age, and would like to get free maternity training. I cannot afford to pay. Can I get into a workhouse infirmary where I could earn a salary and receive a certificate?—*Nurse B.*

Possibly you might get a post as assistant nurse in the lying-in ward of a workhouse. You must then prepare yourself for the examination of the London Obstetrical Society by attending lectures on the theory of maternity nursing. You can get full particulars of the examination from the Society, 20 Hanover Square, W. You have to pay examination fee.

1. Can you kindly tell me where I can get midwifery training without paying a premium? 2. Would a three years' certificate be any advantage?—*A. H. B.*

1. See reply to *Nurse B.* 2. A very great advantage.

*Plague Nursing.*

(236) 1. Do the nurses sent out for plague duty to South Africa belong to the Army Nursing Reserve; and (2) is any special training or qualification required beyond the three years' certificate?—*Bee.*

1. Not Necessarily. 2. No.

I should like to volunteer to nurse the plague either in South Africa, or Hong Kong. Can you tell me where to apply?—*J. R.*

No more nurses are required for plague service in South Africa at present. As to Hong Kong you might write to the Colonial Nursing Association, Imperial Institute, S.W.

*Maternity Nursing.*

(237) 1. I am about to start a midwifery practice. Will you tell me how I can best become known? Should I have engraved on my plate "Miss," "nurse," or my initials only? May I have the initials of the hospital where I was trained on it; and can I wear uniform? 2. What is the usual charge for taking accouchement cases into the house; are references on both sides necessary?—*A. M. R.*

1. Have your professional card with your training and charges on it as widely distributed as possible. Sometimes your local tradesmen will place it where it can be seen. You may put what you like on your plate so long as it is true. Do not call yourself "Nurse" unless you really are a fully trained and certificated nurse. A midwifery training does not make you a "nurse." You can adopt any dress you like. 2. All depends on the class of patient you hope to attract. It is as well to satisfy yourself as to the kind of people you receive into your house.

*Publishing.*

(238) I have written a series of articles on the management of infants, which have appeared in a weekly paper. I would like to publish them in book form. Will you tell me (1) if I have a right to do so; or if they belong to the paper in which they appeared? And (2) how can I set about publishing them?—*L. L.*

Ask the editor who published your articles if you retain the copyright; if so, submit your MSS. to a publisher. You may have to send the book to several before one accepts it.

**Standard Books of Reference.**

**"The Nursing Profession: How and Where to Train." 2s. net; post free 2s. 4d.**

**"Burdett's Official Nursing Directory." 3s. net.; post free, 3s. 4d.**

**"Burdett's Hospitals and Charities." 5s.**

**"The Nurses' Dictionary of Medical Terms." 2s.**

**"Burdett's Series of Nursing Text-Books." 1s. each.**

**"A Handbook for Nurses." (Illustrated.) 5s.**

**"Nursing: Its Theory and Practice." New Edition. 3s. 6d.**

**"Helps in Sickness and to Health." Fifteenth Thousand. 5s.**

**"The Physiological Feeding of Infants." 1s.**

**"The Physiological Nursery Chart." 1s.; post free, 1s. 3d.**

**"Hospital Expenditure: The Commissariat." 2s. 6d.**

**All these are published by THE SCIENTIFIC PRESS, Ltd., and may be obtained through any bookseller or direct from the publishers, 28 and 29 Southampton Street, London, W.C.**

